# Lame Teeth

**Published:** 1909-05-15

**Authors:** R. Ottolengin


### LAME TEETH,

BY R. OTTOLENGIN, M.D.S.

Toothache is the layman's term for any pain originating in or about the teeth, the most severe being pulpitis and acute alveolar abscess. The improper or unskilled treatment of either will leave a tooth in a condition from which it is chronically troublesome, periodically ''sore''; this may be designated a ''lame tooth.'' A correspondent asks three questions in this connection. His first inquiry reads as follows:

''What process of treatment will avoid soreness and tenderness of teeth used as piers in bridgework, after the bridge has been set?''

It will scarcely be the answer expected, to say that roots used as bridge piers should never be sore nor tender after the bridge is set; yet this is true, because no bridge should be permanently attached to roots until the operator is reasonably certain that no soreness or tenderness exists, or will ensue.

Such piers reach the dentist in one of two conditions: either (a) the pulp is alive; or else (b) the pulp has previously died. In the latter case infection may or may not have resulted.

Where the pulp is alive, and presumably healthy, and the dentist intentionally devitalizes it in order to make proper use of the root as a bridge pier, he must under no conditions use arsenic, as this medicament in the past has been responsible for thousands of "lame" teeth. The pulp, therefore, should be removed under pressure anesthesia with cocaine. If this should fail it would be preferable to use nitrous oxid gas, or even ether, chloroform or somnoforme, rather than resort to arsenic. Indeed it is scarcely too much to say that arsenic no longer has a place in dental practice. In connection with cocaine anesthesia and the subsequent removal of the pulp, the strictest asepsis should be maintained. Upon removal of the pulp there is often a hemorrhage and this may occasionally be profuse. It should not be staunched, either by plugging with cotton, or by using adrenalin or other styptics. Any such procedure but invites the formation of a blood clot beyond the apex, and this will account for the tenderness so often reported after the operation of removing living pulps. This blood clot must be absorbed before the tooth can be accounted safe for either filling or bridge, and, of course, during its presence there is danger of infection as such a clot affords fine pabulum for a germ culture.

In the presence of hemorrhage through a tooth root, it is important, therefore, to exhibit patience and even invite bleeding with warm douches and dry cotton tampons, rather than to attempt to dam it back. A root handled in this manner, with perfect asepsis, and provided that all the pulp is removed, will never become sore nor tender.

If the pulp has dried prior to the attempt to utilize the root as a bridge pier, of course it becomes essential to thoroughly cleanse and sterilize the canals, and if an abscess be present, to cure that before attempting to set a bridge. This is not taking into account the splinting of teeth affected by pyorrhea, such procedure being quite the opposite of what is here discussed. In such cases the bridging is done to support the affected roots and the roots are not being used to uphold a bridge.

The second question was: "What is the treatment when there is tenderness after setting a bridge?" The writer was alluding to roots from which living pulps have been intentionally removed. It has been explained above that such roots, properly treated prior to the bridging, will not become tender afterward. Where tenderness does ensue, therefore, the fault is either in the treatment of the root, or else in the bridge itself. Sometimes in assembling, if proper precautions be not taken, the bridge will be shortened by the contracting solder. This makes the bridge bind when setting it, and some operators force it on rather than remedy the defect. The best course is to cut the bridge in half, and resolder, taking precautions against shrinkage. But if the bridge is forced to place, it is evident that the undue stress upon the piers may cause one or both to become tender. But this is the same sort of tenderness which results from wedging for approximal fillings, and if the stress be not too severe the tenderness will pass away, the roots adjusting themselves to the new condition. It is essential, however, to be sure that the occlusion does not contribute to the irritation.

When the tenderness is not traceable to the binding of the bridge, nor to faulty occlusion, and it is known to be due to the pulp removal, the presence of a blood clot at the apex may be suspected. This may sometimes be alleviated by counter irritation, such as an application of capsicum, or of strong iodin (Churchill's). Iodin applid cataphoretically would be even better. Such treatment, however, to be of advantage must be severe enough to produce a blister, and this may cause considerable pain. For this morphine may be exhibited with advantage. But even this may fail, and infection may follow, in which event excision of the end of the root and repeated surgical dressing during healing is the only recourse, always supposing that the bridge can not be removed. The remedies for tenderness after setting a bridge only accentuate the necessity of having the piers absolutely safe before attaching the bridge.

The third question asked was: "What do you do with roots where canals are difficult or impossible of access?"

One of the greatets blunders prevalent among dentists is the attempt to treat multirooted teeth through small openings. Cavities should be so freely cut that ready and direct access may be obtained to all roots needing treatment. After this, even very attenuated canals may be thoroughly explored with patience, an assortment of reliable and very fine broaches, and that most valuable remedy, first suggested by Dr. Emille Schrier, Kalium-natrium (sodium and potassium). The technique of using this preparation has been described many times, and its efficacy is so great that it is extraordinary that it is not more commonly utilized.—*Item of Interest.*

